# Multi-Omics Data Analysis Uncovers Molecular Networks and Gene Regulators for Metabolic Biomarkers

**DOI:** 10.3390/biom11030406

**Published:** 2021-03-10

**Authors:** Su Yon Jung

**Affiliations:** Translational Sciences Section, Jonsson Comprehensive Cancer Center, School of Nursing, University of California, Los Angeles, Los Angeles, CA 90095, USA; sjung@sonnet.ucla.edu

**Keywords:** IGFs/IR axis, multi-omics integration, system biology, molecular pathways, gene network, key drivers

## Abstract

The insulin-like growth factors (IGFs)/insulin resistance (IR) axis is the major metabolic hormonal pathway mediating the biologic mechanism of several complex human diseases, including type 2 diabetes (T2DM) and cancers. The genomewide association study (GWAS)-based approach has neither fully characterized the phenotype variation nor provided a comprehensive understanding of the regulatory biologic mechanisms. We applied systematic genomics to integrate our previous GWAS data for IGF-I and IR with multi-omics datasets, e.g., whole-blood expression quantitative loci, molecular pathways, and gene network, to capture the full range of genetic functionalities associated with IGF-I/IR and key drivers (KDs) in gene-regulatory networks. We identified both shared (e.g., T2DM, lipid metabolism, and estimated glomerular filtration signaling) and IR-specific (e.g., mechanistic target of rapamycin, phosphoinositide 3-kinases, and erb-b2 receptor tyrosine kinase 4 signaling) molecular biologic processes of IGF-I/IR axis regulation. Next, by using tissue-specific gene–gene interaction networks, we identified both well-established (e.g., *IRS1* and *IGF1R*) and novel (e.g., *AKT1*, *HRAS*, and *JAK1*) KDs in the IGF-I/IR-associated subnetworks. Our results, if validated in additional genomic studies, may provide robust, comprehensive insights into the mechanisms of IGF-I/IR regulation and highlight potential novel genetic targets as preventive and therapeutic strategies for the associated diseases, e.g., T2DM and cancers.

## 1. Introduction

The insulin-like growth factors (IGFs)/insulin resistance (IR) axis has been considered one of the major metabolic hormonal pathways that mediate the biologic mechanism of several complex human diseases, such as type 2 diabetes (T2DM), metabolic syndrome, cardiovascular disease, and cancers [1,2,3,4,5,6,7,8,9,10,11]. In particular, abnormal IGF-I levels are related to impaired glucose tolerance (i.e., IR) and to a higher risk of T2DM [12]. The IGFs/IR axis can also be associated with carcinogenesis by aberrantly regulating multiple downstream cell-signaling cascades involved in the promitogenic, proinflammatory, and antiapoptotic signals, thus creating a proneoplastic environment for tumor growth and development in particular cells [6,13,14,15,16,17].

The systemic development of those metabolic cytokines can be influenced by not only environmental [5,18,19] but also genetic factors [20,21,22]. Despite advances in the understanding of genetic variance in relation to those biomarkers, common genetic variants from genomewide association studies (GWASs) explain a moderate proportion of the phenotype variation. For example, GWASs [23] have so far identified more than 83 loci for one or more glycemic traits, together explaining about 20% of the genetic heritability [24]; this suggests that more than two thirds of heritability is still to be discovered.

Conventional GWASs examine single genetic markers one at a time, leading to a lack of statistical power due to multiple testing corrections. Thus, even very large GWASs may not be adequately powered to identify genetic variants with small effect sizes and low allele frequencies, suggesting the need for a group-level analysis of genes/single-nucleotide polymorphisms (SNPs) in their biologic pathways [25,26]. Further, GWASs are not designed to evaluate the tissue-specific gene–gene interactions that can play a critical role in accounting for the missing heritability. Further, the genetic loci identified by GWASs often have unclear functionality; thus, the molecular mechanism underlying the effects of genetic loci on a given phenotype is not well characterized. Various molecular pathway– and gene network–based strategies using GWAS findings have been developed [27,28] showing that they are powerful sufficiently to capture the missing heritability of quantitative phenotypes [29,30]. The biologic pathway–based approach can also detect the functionality of the genes in enriched molecular signaling cascades. In addition, tissue-specific analyses of gene regulatory networks can capture the causal regulatory relationships between genes under different pathophysiological conditions and identify key drivers (KDs) as important hub genes regulating subnetwork genes in a particular enriched pathway.

**Figure 1:**
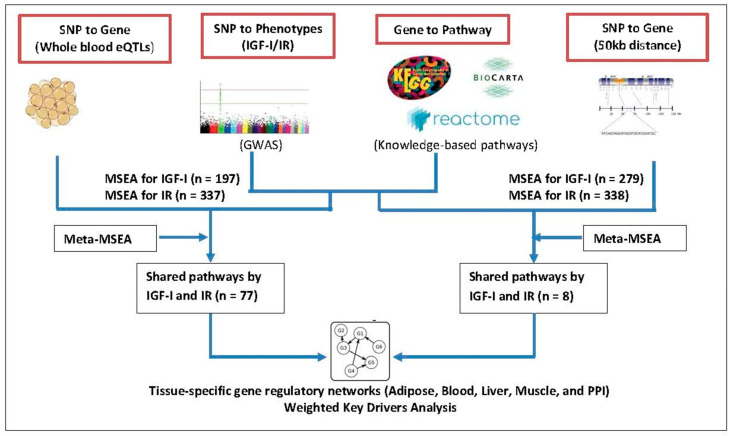
Schematic diagram of the study. (eQTL, expression quantitative trait loci; IGF-I, insulin-growth factor-I; IR, insulin resistance; MSEA, marker-set enrichment analysis; SNP, single nucleotide polymorphism).

In this study, we applied an integrative genomics approach (Figure 1) that combines our previous GWAS findings for IGF-I and IR [31] with functional genomics data, including whole-blood expression quantitative loci (eQTLs, for revealing functional regulation of gene expression); molecular pathways; and data-driven gene networks to provide gene–gene (G × G) interaction information from the key tissues involved in the IGF-I/IR axis. Our study, by integrating genetic loci with multi-omics datasets, may unravel the full range of genetic functionalities and their regulation (from strong to subtle) in the gene networks, thus providing comprehensive novel insights into the molecular mechanisms of IGF-I/IR and potential preventive and therapeutic strategies for IGF-I/IR–associated diseases.

## 2. Materials and Methods

### 2.1. GWAS Data for IGF-I and IR Phenotypes

Detailed study rationale, design, genotyping, and summarized genomic statistics have been described previously [32,33]. Briefly, the Women’s Health Initiative (WHI) Harmonized and Imputed GWAS was designed to contribute a joint imputation and harmonization effort for GWASs within the WHI Clinical Trials and Observational Studies. WHI study participants include postmenopausal women enrolled at more than 40 clinical centers nationwide from 1 October 1993, through 31 December 1998. Eligible women were 50–79 years old, postmenopausal, expected to reside near the clinical centers for at least 3 years after enrollment, and able to provide written informed consent. The WHI Harmonization and Imputation Studies involved datasets from 6 GWASs: *MOPMAP[AS264]*; *GARNET*; *GECCO-CYTO*; *GECCO-INIT*; *HIPFX*; and *WHIMS*. By using those 6 GWASs, our previous GWAS [31] included 11,794 women who reported their race or ethnicity as non-Hispanic white; we conducted a GWAS meta-analysis of gene–environmental interaction (G × E) for IGF-I/IR phenotypes. Our study was approved by the institutional review boards of each participating clinical center of the WHI and by the University of California, Los Angeles.

### 2.2. Genotyping and IGF-I/IR Phenotypes

Genomewide genotyping of the WHI Harmonized and Imputed GWASs was performed, followed by normalization of the genotype calls to the reference panel GRCh37 and genotype imputation using 1000 genome reference panels [33]. The minimum cutoff of allele frequency across GWASs averaged 1.5%. Our previous GWAS analyzed 18,717,781 common autosomal SNPs, combining the GWA results across 6 GWASs, assuming a fixed-effect model by adjusting for age and 10 genetic principal components. The phenotypes examined included fasting serum levels of bioavailable IGF-I and homeostasis model assessment for IR (HOMA-IR, estimated as glucose (unit: mg/dL) × insulin (unit: μIU/mL)/405 [34]).

### 2.3. Mergeomics

We used Mergeomics [35], a robust computational pipeline, to identify molecular pathways, gene networks, and key regulators via integration of multi-omics datasets such as statistical summaries of phenotype associations and molecular networks. Mergeomics has demonstrated superior performance to that of other gene-set enrichment analytic methods [35]. In particular, it less likely to be affected by the heterogeneity between independent datasets from different studies, thus detecting relatively robust biological signals across data types and studies.

#### 2.3.1. Mapping SNPs to Genes

We used 2 different mapping methods to connect SNPs to the potential target genes and generated 2 sets (one per mapping method) of SNP–gene maps. First, a standard chromosomal distance–based approach with global use of 50 kb gene boundaries was used to generate a distance-based map within 50 kb of the gene region. Next, an eQTL-based mapping approach was used. Because gene expression levels can be considered quantitative traits in GWASs, determining the expression SNPs (eSNPs) associated with the gene expression (i.e., eQTLs) can capture the potential functional relationship between SNPs and expressed genes. Further, the eSNPs within the eQTLs are tissue specific. We used the whole-blood eQTLs and selected *cis*-eSNPs (within 1 Mb of the gene region at a false discovery rate (FDR) < 0.05) to find mechanistic clues in peripheral blood mononuclear cells where the gene expression intersected the IGF/IR-eSNPs. Linkage disequilibrium (LD) structure was corrected by keeping SNPs that have strong associations with phenotypes in LD (R^2^ > 0.5).

#### 2.3.2. Marker-Set Enrichment Analysis (MSEA)

We used knowledge-based pathways that include 1827 canonical pathways from the Reactome, Biocarta, and the Kyoto Encyclopedia of Genes and Genomes (KEGG) databases [36,37]. To uncover the gene sets involved in the metabolic and signaling pathways, we used the MSEA in the Mergeomics package, which is one of the well-established methods. Using the MSEA, we tested each pathway for enrichment of genes for IGF-I/IR phenotypes on the basis of modified chi-square statistics [35,38] which adapt the summarized cutoff (not a single GWAS *p* value) over a range of quantiles for marker selections. A FDR < 0.05 was considered statistically significant. To capture the core gene sets from redundant pathways across the 2 phenotypes, we further conducted the Meta-MSEA analysis in Mergeomics to perform a meta-analysis.

#### 2.3.3. Tissue-Specific Gene Regulatory Networks and Weighted KD Analysis

The next step in the Mergeomics pipeline was to perform KD analysis to identify key regulators involved in the statistically significant pathways (i.e., FDR < 0.05) from predefined gene regulatory networks. For this analysis, we employed (i) Bayesian gene regulatory networks constructed from genetic and gene expression data of blood and adipose, liver, and muscle tissues and (ii) protein–protein interaction networks (PPIs) [39,40]. We performed a weighted KD analysis [35,41,42] to detect KDs whose neighborhood network presented significant enrichment on the basis of modified chi-square statistics [35,38] at FDR < 0.05. The top KDs thus showed high network enrichment for the genes in pathways.

## 3. Results

### 3.1. Phenotype-Specific and Common Pathways Shared by IGF-I and IR

We first conducted phenotype-specific MSEA for IGF-I and IR and revealed a tissue-specific regulatory mechanism. Specifically, among the significant pathways (FDR < 0.05) for the enrichment of gene sets for IGF-I, 59 pathways overlapped between distance-based (20% of 279 gene sets) and eQTL-based mapping (29% of 197 gene sets) (Figure S1). These included T2DM, glycosaminoglycan and lipoprotein metabolism, and signaling by estimated glomerular filtration rate (EGFR) as top pathways. For the IR-specific pathways, 100 pathways from the significantly enriched pathways overlapped between distance-based (26% of 388 gene sets) and eQTL-based mapping (30% of 337 gene sets) (Figure S2). Some of the top pathways were similar to those from IGF-I-specific pathways, such as T2DM, corticosteroid mechanism, mechanisms of lipids and lipoproteins, fatty acid, and triglycerides (TG), and EGFR signaling, whereas IR-unique pathways included mechanistic target of rapamycin (mTOR) signaling, phosphoinositide 3-kinase (PI3K) subunit p85, and erb-b2 receptor tyrosine kinase 4 (ERBB4) signaling. Pathways from the two phenotypes also included transcription function (e.g., genetic/nuclear receptor transcription and metabolism of RNA, mRNA, and noncoding RNA), which was not surprising given that the IGFs/IR axis functions as a mitogen.

**Figure 2:**
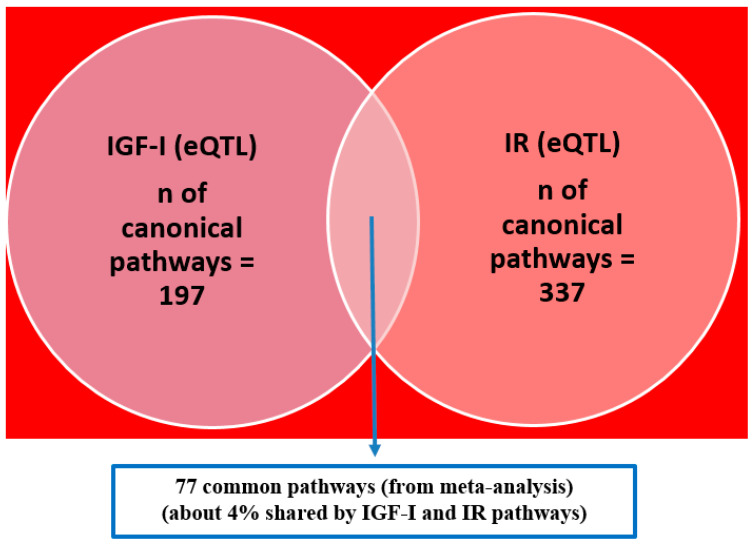
Comparison of significant pathways (false discovery rate [FDR] < 0.05) between insulin-like growth factor-I (IGF-I) and insulin resistance (IR) phenotypes (IGF-I/IR, expression quantitative trait loci [eQTL]-based mapping to genes).

Next, we performed a Meta-MSEA between IGF-I and IR in distance-based and eQTL-based mapping to identify shared pathways enriched by gene sets for both phenotypes. Distance mapping–based Meta-MSEA (Figure S3) showed 8 (3%) pathways shared by IGF-I and IR, including known IGF/IR axis pathways (e.g., gene control of body mass index and lipid metabolism) as well as lesser-known pathways, including mucopolysaccharidosis type III, Notch-1 heterodimerization domain mutation in cancer, and serotonin neurotransmitter release cycle. For the eQTL mapping–based Meta-MSEA, 77 pathways (4%) were shared by the two phenotypes (Figure 2 and Table S1). The shared pathways included general cellular pathways (e.g., oxidative phosphorylation, calcium signaling, and iron uptake and transport) and, notably, involved glucose metabolism–unique pathways, such as glycosaminoglycan biosynthesis, glucagon signaling in metabolic regulation, and insulin receptor recycling.

Further, six pathways were found to be shared by both distance- and eQTL based–mapping types for IGF-I and IR (Figure S4), all of which overlapped with the pathways from the Meta-MSEA of eQTL mapping–based IGF-I/IR. Those shared pathways included cellular-based pathways, such as heparan sulfate/heparin biosynthesis and mitochondrial protein import, and well-known IGF-I/IR axis pathways, including T2DM, lipoprotein metabolism, and EGFR signaling (Figure S4) As described, the Meta-MSEA analysis of eQTL-based mapping pathways for IGF-I and IR, compared with the analysis of the distance-based mapping pathways, yielded more informative pathways. This suggests that functional eSNPs associated with gene expression within whole blood better captured the mechanisms regulating serum IGF-I/IR, thus leading us to focus on the eQTL mapping–based IGF-I/IR for further analysis.

### 3.2. Putative Key Regulatory Genes (i.e., KDs) for the IGF-I/IR–Associated Pathways

**Figure 3:**
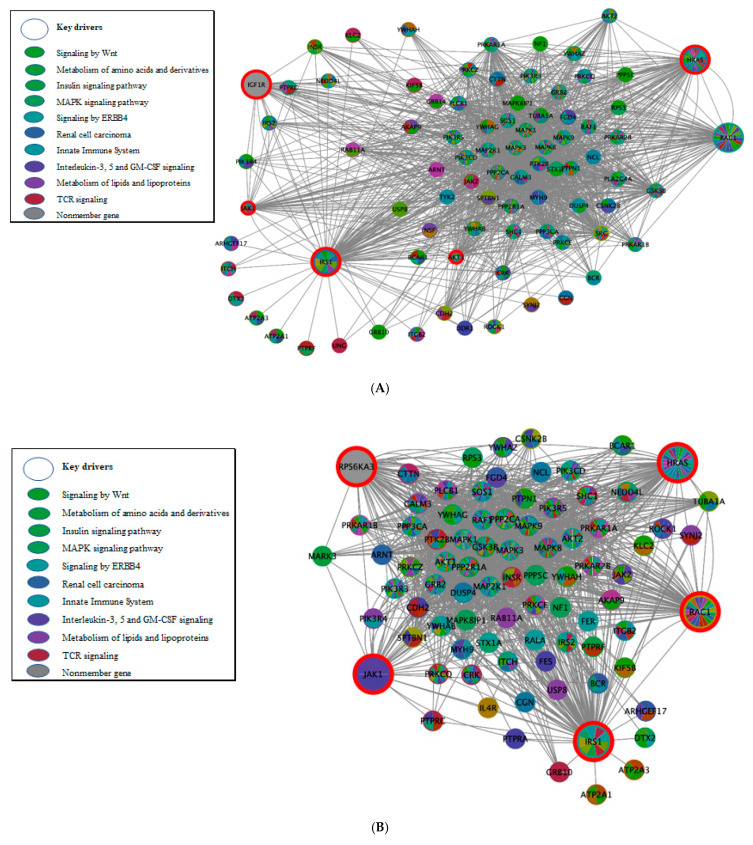
PPI-specific gene-regulatory networks of top 5 KDs in IGF-I and IR (eQTL mapping). (eQTL, expression quantitative trait loci; IGF-I, insulin-growth factor-I; IR, insulin resistance; KD, key drivers; PPI, protein to protein interaction network; T2DM, type 2 diabetes; wKDA, weighted KD analysis). The bigger nodes with red outlines are top KDs in the enriched pathway obtained from wKDA. The subnetworks of the KDs are indicated by different colors according to their differences in canonical functions. (**A**) T2DM (module M19708)–specific KDs and subnetworks (from the meta-analysis of IGF-I and IR); (**B**) insulin signaling pathway (module M18155)–specific KDs and subnetworks (from IR eQTLs).

By using the 77 shared pathways identified by eQTL mapping–based IGF-I and IR, we next performed KD analysis to detect within the G × G interaction networks important hub genes (i.e., KDs) whose neighborhoods are overrepresented with the genes in the IGF-I/IR pathways. In addition to PPIs, we obtained tissue-specific KDs from blood and adipose, liver, and muscle tissues because they play a key role in regulating the IGF-I/IR axis. Among 25 shared subnetworks enriched with KDs from tissues and PPIs (Table S2), we detected two subnetworks (Table 1) that overlapped with the six pathways shared by distance- and eQTL mapping–based IGF-I/IR: T2DM and fatty-acid, TG, and ketone-body metabolism. Interestingly, the KDs of those two subnetworks were identified only from the PPI network. In particular, the top five KDs of the T2DM subnetwork were *IRS1*, *HRAS*, *JAK1*, *IGF1R*, and *AKT1* (Table 1). Further, they are interrelated with the neighboring subnetworks of insulin, mitogen-activated protein kinase (MAPK), and ERBB4 signaling; renal-cell carcinogenetic mechanism; innate immune and interleukin signaling; and lipid metabolism (Figure 3A). In addition, the top five KDs of the subnetwork for fatty-acid, TG, and ketone-body metabolism were *MED24*, *MED15*, *MED6*, *MED1*, and *CDK8* (Table 1).

Further, HOMA-IR estimation has been used as a good proxy for IR. Therefore, we additionally focused on the IR phenotype to reveal associated molecular mechanisms by identifying KDs in the subnetworks enriched by gene sets for the eQTL mapping based–IR. Of the 95 subnetworks involved (Table S3), six selected subnetworks are shown in Table 2: adipokine; insulin, MAPK, and EGFR signaling; innate immune system; and fatty acid metabolism. Particularly, the top five KDs of the insulin-signaling subnetwork were *IRS1*, *HRAS*, *RAC1*, *JAK1*, and *RPS6KA3* (Table 2), similar to the aforementioned top five KDs of the T2DM subnetwork. Thus, their interrelated neighborhood subnetworks were also similar to those connected to T2DM (Figure 3B).

## 4. Discussion

A growing number of population-based genomic studies [27,43,44] support that the comprehensive examination of multiple genes in molecular pathways and in G × G interaction networks, compared to the individual gene-level approach, contributes more to revealing the underlying mechanisms of quantitative phenotypes and complex diseases. To detect the biologic mechanism that may not be obvious from the individual top GWAS hits alone, we integrated our previous GWAS data with eQTLs, knowledge-driven biologic pathways, and gene-regulatory networks and found diverse sets of genes within the biologic pathways, associated with individual IGF-I and IR and across these phenotypes. Further, our tissue-specific gene-network analyses revealed both well-known and novel KDs in the IGF-I/IR biological processes. Our findings thus offer robust and comprehensive insights into the molecular regulation of the IGF-I/IR metabolism, which may have been missed without systematic genomics approaches.

In particular, the shared pathways we identified across the phenotypes in both distance- and eQTL based–mapping included T2DM, lipoprotein/TG/fatty acid metabolism, and EGFR signaling. T2DM [1,2,3,4] and lipid metabolism [45] are linked well to the IGF-I/IR axis. In regard to the lipid profile, previous in vivo and in vitro studies [46,47,48,49] indicated that IGF-I, IGF binding protein 3, insulin receptor, and IGF-I receptor (IGF-IR) correlated positively with TG, the TG/high-density lipoprotein (HDL) ratio, and fatty acid synthesis, inducing IR. Further, high levels of TG, high levels of low-density lipoprotein, and low levels of HDL were found in patients with T2DM [50,51,52]. One unique pathway involved, EGFR signaling, has been implicated in glucose homeostasis by regulating beta-cell proliferation in response to increased metabolic demand [53]. Notably, EGFR signaling is associated with IGF-IR expression and IGF-I secretion in cancer cells [54,55], contributing to cancer cell growth and poor survival; thus, dual targeting at EGFR and the IGF/IR axis has been suggested to be a promising therapeutic strategy for overcoming drug-acquired resistance in several cancer types, such as lung adenocarcinoma, head and neck squamous cell and colorectal carcinomas, and glioblastoma [55,56,57,58].

Next, because hundreds of genes are involved in the identified biologic pathways, we used the G × G interaction networks and identified key regulators of those significant pathways to uncover novel regulatory mechanisms and prioritize the genes that are involved. For shared pathways across the phenotypes and IR-specific pathways, we detected repeated but meaningful PPI-specific subnetworks, such as T2DM, adipokin, insulin, and EGFR signaling and, additionally, their neighboring subnetworks, including MAPK, innate immune system, ERBB4, and renal-cell carcinogenetic mechanism. In particular, the *ERBB4* gene is a tyrosine-protein kinase that plays an essential role as a cell surface receptor for the epidermal growth factor family, mediating activation of the MAPK/PI3K/serine/threonine-specific protein kinase 1 (AKT1) [59,60]. The ERBB4 signaling, in addition to PIK3/AKT, has been suggested as a potential target for treatment of malignant bone tumors [61]. Further, *ERBB4* genetic variants are associated with T2DM and type 1 diabetes nephropathy [62,63]. Taken together, ERBB4 signaling adjacent to the T2DM and renal cell carcinogenetic mechanism subnetworks can be studied as potential promising targets and biomarkers for T2DM-associated renal cell carcinoma.

Of the top five KDs detected in relation to the T2DM subnetwork, two KDs (*IRS1* and *IGF1R*) are known regulators for T2DM, so they have served as effective drug targets according to the DrugBank database [64]. Further, the three remaining KDs identified in the T2DM subnetwork include *AKT1*, *HRAS*, and *JAK1*, two (*HRAS*, and *JAK1*) of which were also found to be top KDs in the insulin signaling network. Those three KDs are interrelated with other diabetes genes and are involved in the downstream pathways such as the interleukin-6/signal transducer and the activator of the transcription 3 (STAT3) and immune/inflammation responses [65,66,67,68,69,70,71]; thus, they have implications as novel targets for IGF/IR-associated disorders, including T2DM.

Our GWAS database may not capture the full array that covers unknown biology in relation to the IGF-I/IR axis. We also did not perform directional analyses. Our approach did not detect epistatic interactions among genetic factors. Further, because our study was restricted to non-Hispanic white postmenopausal women, the generalizability of our findings to other populations is limited. Nevertheless, our study has detected well-established pathways in relation to the phenotypes and several KDs that have been targeted by FDA-approved drugs, indicating that our integrative multi-omics data approach was robust and powerful. Further, consistent with the findings of other studies [26,38], the KDs we identified in our study were not the top GWAS hits owing to evolutionary constraints [72,73]. However, because those KDs have central properties in the networks, exerting strong effects on phenotype regulation and related-disease risk/progression, they can be considered to be better candidates for drug targets and biomarkers.

## 5. Conclusions

Our study identified both shared (e.g., T2DM, lipid metabolism, and EGFR signaling) and distinct (e.g., mTOR, PI3K, and ERBB4 signaling for IR) molecular pathways underlying IGF-I/IR axis regulation. The tissue-specific gene regulatory networks revealed several key drivers, both well-established (e.g., *IRS1* and *IGF1R*) and novel (e.g., *AKT1*, *HRAS*, and *JAK1*), for the involved biologic mechanisms. Our findings warrant further validation in an independent large genetic and mechanistic dataset. Nevertheless, our study may contribute to better capturing of the potential genetic targets for regulating the IGFs/IR axis as preventive and therapeutic strategies for the associated diseases such as T2DM and cancers.

## Figures and Tables

**Table 1:** MSEA meta-analysis of IGF-I and IR pathways (eQTL-based mapping to genes) and corresponding tissue-specific network key drivers (two modules are presented, being shared by IGF-I and IR pathways on the basis of 50-kb distance and eQTL-mapping).

		Module Size of PPI (*n* of Genes)	Top 5 Key Drivers				
Module	Description		Adipose	Blood	Liver	Muscle	PPI
M19708	Type 2 diabetes mellitus	17	N/A	N/A	N/A	N/A	*IRS1* *, *HRAS*, *JAK1*, *IGF1R*, *AKT1*
rctm0415	Fatty acid, triacylglycerol, and ketone body metabolism	46	N/A	N/A	N/A	N/A	*MED24* *, *MED15* *, *MED6* *, *MED1*, *CDK8*

eQTL, expression quantitative trait loci; IGF-I, insulin-growth factor-I; IR, insulin resistance; MSEA, marker-set enrichment analysis; N/A, not available; PPI, protein to protein interaction network. * Member gene of the particular pathway in PPI-specific gene-regulatory network analysis.

**Table 2:** Selected IR pathways (eQTL-based mapping to genes) from MSEA and corresponding tissue-specific network key drivers.

Module	Description	Module Size (*n* of Genes)	Top 5 Key Drivers				
			Adipose	Blood	Liver	Muscle	PPI
M10462	Adipocytokine signaling pathway	N/A **, N/A ¶, N/A ¥, N/A †, 33 §	N/A	N/A	N/A	N/A	*GSK3B*, *FRAP1*, *HSP90AA2*, *PDPK1*, *IKBKB*
M10792	MAPK signaling pathway	N/A **, N/A ¶, N/A ¥, N/A †, 63 §	N/A	N/A	N/A	N/A	*MAPK9* *, *MAPK8* *, *MAP2K1* *, *MAP3K11* *, *MAPK10*
M18155	Insulin signaling pathway	N/A **, N/A ¶, N/A ¥, N/A †, 58 §	N/A	N/A	N/A	N/A	*IRS1* *, *HRAS* *, *RAC1*, *JAK1*, *RPS6KA3*
M699	Fatty acid metabolism	30 **, N/A ¶, 30 ¥, 28 †, N/A §	*HADHB* *, *ACADVL* *, *ECHS1* *, *ETFDH*	N/A	*HADH* *, *ACADM* *	*HADHB* *	N/A
rctm0354	EGFR downregulation	N/A **, N/A ¶, N/A ¥, N/A †, 15 §	N/A	N/A	N/A	N/A	*EGF* *, *UBA52* *, *EGFR*, *UBC*, *RPS27A*
rctm0591	Innate immune system	251 **, N/A ¶, 252 ¥, 223 †, 282 §	*LAT2* *, *PTPN6*, *NCKAP1L*, *IL10RA*, *IRF5*	N/A	*TYROBP* *, *NCKAP1L*, *RAC2*, *NCF2*, *IGSF6*	*AK014135*, *COTL1*	*GRB2* *, *MAPKAPK2*, *RAP2A*, *FRK*, *C1QC*

EGFR, estimated glomerular filtration rate; eQTL, expression quantitative trait loci; IR, insulin resistance; MAPK, mitogen-activated protein kinase; MSEA, marker-set enrichment analysis; N/A, not available; PPI, protein to protein interaction network. ** Number of genes in adipose-specific network pathways. ¶ Number of genes in blood-specific network pathways. ¥ Number of genes in liver-specific network pathways. † Number of genes in muscle-specific network pathways. § Number of genes in PPI-based network pathways. * Member gene of the particular pathway in tissue-specific gene-regulatory network analysis.

## Data Availability

The data that support the findings of this study are available in accordance with policies developed by the NHLBI and WHI in order to protect sensitive participant information and approved by the Fred Hutchinson Cancer Research Center, which currently serves as the IRB of record for the WHI. Data requests may be made by emailing to helpdesk@WHI.org.

## Supplementary Materials

The following are available online at https://www.mdpi.com/2218-273X/11/3/406/s1, Figure S1: Comparison of significant pathways (false discovery rate [FDR] < 0.05) for insulin-like growth factor-I (IGF-I) phenotype between 50-kb distance–based and expression quantitative trait loci [eQTL]–based mapping to genes, Figure S2: Comparison of significant pathways (false discovery rate [FDR] < 0.05) for insulin resistance (IR) phenotype between 50-kb distance–based and expression quantitative trait loci [eQTL]–based mapping to genes, Figure S3: Comparison of significant pathways (false discovery rate [FDR] < 0.05) between insulin-like growth factor-I (IGF-I) and insulin resistance (IR) phenotypes (IGF-I/IR, 50-kb distance–based mapping to genes), Figure S4: Comparison of significant pathways (false discovery rate [FDR] < 0.05) between insulin-like growth factor-I (IGF-I) and insulin resistance (IR) phenotypes (IGF-I/IR, 50-kb distance–based and expression quantitative trait loci [eQTL]–based mapping to genes; yellow-highlighted pathways are significant [FDR < 0.05] in the marker-set enrichment meta-analysis of IGF-I-eQTL and IR-eQTL), Table S1: Meta-MSEA analysis of IGF-I and IR pathways (IGF-I/IR, eQTL-based mapping to genes; pathways arranged by ascending FDR), Table S2: IGF-I and IR pathways (eQTL-based mapping to genes) from the MSEA meta-analysis and corresponding tissue-specific network key drivers, Table S3: IR pathways (eQTL-based mapping to genes) from MSEA and corresponding tissue-specific network key drivers.
