# Supplementary material for: Multi-Omics Data Analysis Uncovers Molecular Networks and Gene Regulators for Metabolic Biomarkers
Source: Biomolecules. 2021 Mar 10;11(3):406. doi: 10.3390/biom11030406 (PMC8001935; doi:10.3390/biom11030406)
Supplement: Supplementary file 1 [file biomolecules-11-00406-s001.zip › Supple Figure 1.docx]

Figure S1. Comparison of significant pathways (false discovery rate [FDR] < 0.05) for insulin-like growth factor-I (IGF-I) phenotype between 50-kb distance–based and expression quantitative trait loci [eQTL]–based mapping to genes

**Fifty-nine common pathways**

**(about 20% of IGF-50 kb and 29% of IGF-eQTL)**

| **Common Pathways** | | | |
| --- | --- | --- | --- |
| M13486 | Huntington's disease | rctm0524 | HIV Transcription Elongation |
| M14091 | Olfactory transduction | rctm0526 | HIV elongation arrest and recovery |
| M1840 | Glutathione metabolism | rctm0527 | HS-GAG biosynthesis |
| M19540 | Oxidative phosphorylation | rctm0532 | Heparan sulfate/heparin (HS-GAG) metabolism |
| M19708 | Type 2 diabetes mellitus | rctm0640 | Late Phase of HIV Life Cycle |
| M3494 | Thrombin signaling and protease-activated receptors | rctm0648 | Lipoprotein metabolism |
| M6382 | Regulation of autophagy | rctm0663 | MPS I - Hurler syndrome |
| M7014 | PKC-catalyzed phosphorylation of inhibitory phosphoprotein of myosin phosphatase | rctm0664 | MPS II - Hunter syndrome |
| M7151 | Selenoamino acid metabolism | rctm0665 | MPS IIIA - Sanfilippo syndrome A |
| M7272 | Parkinson's disease | rctm0666 | MPS IIIB - Sanfilippo syndrome B |
| M7330 | Glycosaminoglycan biosynthesis - heparan sulfate | rctm0667 | MPS IIIC - Sanfilippo syndrome C |
| rctm0033 | Abortive elongation of HIV-1 transcript in the absence of Tat | rctm0668 | MPS IIID - Sanfilippo syndrome D |
| rctm0066 | Activation of NMDA receptor upon glutamate binding and postsynaptic events | rctm0669 | MPS IV - Morquio syndrome A |
| rctm0171 | Biological oxidations | rctm0670 | MPS IV - Morquio syndrome B |
| rctm0177 | Botulinum neurotoxicity | rctm0671 | MPS IX - Natowicz syndrome |
| rctm0205 | CaM pathway | rctm0672 | MPS VI - Maroteaux-Lamy syndrome |
| rctm0208 | Calmodulin induced events | rctm0673 | MPS VII - Sly syndrome |
| rctm0224 | Cell-Cell communication | rctm0709 | Mitochondrial Protein Import |
| rctm0242 | Citric acid cycle (TCA cycle) | rctm0711 | Mitochondrial tRNA aminoacylation |
| rctm0368 | Elastic fibre formation | rctm0728 | Mucopolysaccharidoses |
| rctm0372 | Elongation arrest and recovery | rctm0753 | NOD1/2 Signaling Pathway |
| rctm0425 | Formation of HIV elongation complex in the absence of HIV Tat | rctm0849 | Pausing and recovery of HIV elongation |
| rctm0426 | Formation of HIV-1 elongation complex containing HIV-1 Tat | rctm0850 | Pausing and recovery of Tat-mediated HIV elongation |
| rctm0427 | Formation of RNA Pol II elongation complex | rctm0952 | RNA Polymerase II Transcription Elongation |
| rctm0433 | Formation of the Early Elongation Complex | rctm1110 | Signal regulatory protein (SIRP) family interactions |
| rctm0435 | Formation of the HIV-1 Early Elongation Complex | rctm1114 | Signaling by EGFR |
| rctm0495 | Generic Transcription Pathway | rctm1115 | Signaling by EGFR in Cancer |
| rctm0513 | Glycosaminoglycan metabolism | rctm1251 | Tat-mediated HIV elongation arrest and recovery |
| rctm0516 | Golgi Associated Vesicle Biogenesis | rctm1252 | Tat-mediated elongation of the HIV-1 transcript |
|  |  | rctm1374 | YAP1- and WWTR1 (TAZ)-stimulated gene expression |
