# Supplementary material for: Multi-Omics Data Analysis Uncovers Molecular Networks and Gene Regulators for Metabolic Biomarkers
Source: Biomolecules. 2021 Mar 10;11(3):406. doi: 10.3390/biom11030406 (PMC8001935; doi:10.3390/biom11030406)
Supplement: Supplementary file 1 [file biomolecules-11-00406-s001.zip › Supple Figure 2.docx]

Figure S2. Comparison of significant pathways (false discovery rate [FDR] < 0.05) for insulin resistance (IR) phenotype between 50-kb distance–based and expression quantitative trait loci [eQTL]–based mapping to genes

`

**One hundred common pathways**

**(about 26% of IR-50 kb and 30% of IR-eQTL)**

| **Common Pathways** | | | |
| --- | --- | --- | --- |
| M10066 | Corticosteroids and cardioprotection | rctm0477 | GPVI-mediated activation cascade |
| M10082 | TNFR2 Signaling Pathway | rctm0514 | Glycosphingolipid metabolism |
| M11650 | Nitric Oxide Signaling Pathway | rctm0523 | HIV Life Cycle |
| M11673 | Biosynthesis of unsaturated fatty acids | rctm0527 | HS-GAG biosynthesis |
| M11835 | Valine, leucine and isoleucine degradation | rctm0533 | Hexose transport |
| M13515 | Mismatch repair | rctm0589 | Initial triggering of complement |
| M1519 | Endocytosis | rctm0598 | Integration of energy metabolism |
| M1547 | Control of skeletal myogenesis by HDAC and calcium/calmodulin-dependent kinase (CaMK) | rctm0647 | Lipid digestion, mobilization, and transport |
| M16120 | How does salmonella hijack a cell | rctm0648 | Lipoprotein metabolism |
| M16473 | Aldosterone-regulated sodium reabsorption | rctm0682 | Metabolism of RNA |
| M16476 | Cell adhesion molecules (CAMs) | rctm0683 | Metabolism of amino acids and derivatives |
| M16563 | mTOR Signaling Pathway | rctm0686 | Metabolism of lipids and lipoproteins |
| M1940 | Regulation And Function Of ChREBP in Liver | rctm0687 | Metabolism of mRNA |
| M19708 | Type 2 diabetes mellitus | rctm0689 | Metabolism of non-coding RNA |
| M19895 | Nicotinate and nicotinamide metabolism | rctm0709 | Mitochondrial Protein Import |
| M2668 | beta-Alanine metabolism | rctm0740 | N-glycan trimming in the ER and Calnexin/Calreticulin cycle |
| M4086 | Propanoate metabolism | rctm0750 | NGF signalling via TRKA from the plasma membrane |
| M4361 | Proximal tubule bicarbonate reclamation | rctm0770 | Negative regulation of FGFR signaling |
| M4791 | Regulation of eIF4e and p70 S6 Kinase | rctm0798 | Nuclear Receptor transcription pathway |
| M5290 | Role of MEF2D in T-cell Apoptosis | rctm0817 | Other semaphorin interactions |
| M5291 | Role of PI3K subunit p85 in regulation of Actin Organization and Cell Migration | rctm0840 | PLC-gamma1 signalling |
| M766 | Glycine, serine and threonine metabolism | rctm0842 | PLCG1 events in ERBB2 signaling |
| rctm0038 | Activated NOTCH1 Transmits Signal to the Nucleus | rctm0845 | PTM: gamma carboxylation, hypusine formation and arylsulfatase activation |
| rctm0059 | Activation of Gene Expression by SREBP (SREBF) | rctm0876 | Platelet sensitization by LDL |
| rctm0111 | Amyloids | rctm0883 | Post-Elongation Processing of Intron-Containing pre-mRNA |
| rctm0118 | Antigen processing: Ubiquitination & Proteasome degradation | rctm0884 | Post-Elongation Processing of Intronless pre-mRNA |
| rctm0181 | Budding and maturation of HIV virion | rctm0885 | Post-Elongation Processing of the Transcript |
| rctm0246 | Class I MHC mediated antigen processing & presentation | rctm0897 | Prefoldin mediated transfer of substrate to CCT/TriC |
| rctm0249 | Classical antibody-mediated complement activation | rctm0903 | Processing of Capped Intron-Containing Pre-mRNA |
| rctm0252 | Cleavage of Growing Transcript in the Termination Region | rctm0904 | Processing of Capped Intronless Pre-mRNA |
| rctm0274 | Cooperation of Prefoldin and TriC/CCT in actin and tubulin folding | rctm0933 | Pyruvate metabolism and Citric Acid (TCA) cycle |
| rctm0277 | Creation of C4 and C2 activators | rctm0956 | RNA Polymerase II Transcription Termination |
| rctm0294 | DAG and IP3 signaling | rctm0987 | Regulation of Cholesterol Biosynthesis by SREBP (SREBF) |
| rctm0297 | DARPP-32 events | rctm1062 | Role of LAT2/NTAL/LAB on calcium mobilization |
| rctm0310 | Deadenylation-dependent mRNA decay | rctm1063 | Role of phospholipids in phagocytosis |
| rctm0344 | Downstream Signaling Events Of B Cell Receptor (BCR) | rctm1095 | Scavenging of Heme from Plasma |
| rctm0345 | Downstream TCR signaling | rctm1109 | Signal attenuation |
| rctm0347 | Downstream signaling of activated FGFR | rctm1114 | Signaling by EGFR |
| rctm0354 | EGFR downregulation | rctm1115 | Signaling by EGFR in Cancer |
| rctm0355 | EGFR interacts with phospholipase C-gamma | rctm1117 | Signaling by ERBB4 |
| rctm0392 | FCERI mediated Ca+2 mobilization | rctm1118 | Signaling by FGFR |
| rctm0394 | FCERI mediated NF-kB activation | rctm1119 | Signaling by FGFR in disease |
| rctm0411 | Factors involved in megakaryocyte development and platelet production | rctm1146 | Signaling by SCF-KIT |
| rctm0412 | Fanconi Anemia pathway | rctm1155 | Signalling by NGF |
| rctm0414 | Fatty Acyl-CoA Biosynthesis | rctm1228 | TCR signaling |
| rctm0415 | Fatty acid, triacylglycerol, and ketone body metabolism | rctm1266 | The citric acid (TCA) cycle and respiratory electron transport |
| rctm0417 | Fc epsilon receptor (FCERI) signaling | rctm1335 | Triglyceride Biosynthesis |
| rctm0418 | Fcgamma receptor (FCGR) dependent phagocytosis | rctm1388 | mRNA 3'-end processing |
| rctm0439 | Formation of tubulin folding intermediates by CCT/TriC | rctm1398 | mRNA Splicing - Minor Pathway |
| rctm0450 | G alpha (z) signalling events | rctm1415 | snRNP Assembly |
