# Supplementary material for: Multi-Omics Data Analysis Uncovers Molecular Networks and Gene Regulators for Metabolic Biomarkers
Source: Biomolecules. 2021 Mar 10;11(3):406. doi: 10.3390/biom11030406 (PMC8001935; doi:10.3390/biom11030406)
Supplement: Supplementary file 1 [file biomolecules-11-00406-s001.zip › Supple Figure 3.docx]

Figure S3. Comparison of significant pathways (false discovery rate [FDR] < 0.05) between insulin-like growth factor-I (IGF-I) and insulin resistance (IR) phenotypes (IGF-I/IR, 50-kb distance–based mapping to genes)

**Eight common pathways (from meta-analysis)**

**(about 3% shared by IGF-I and IR pathways)**

| Pathway | Description | FDR < 0.05 |
| --- | --- | --- |
| rctm0665 | MPS IIIA - Sanfilippo syndrome A | 1.88E-30 |
| rctm1103 | Serotonin Neurotransmitter Release Cycle | 1.01E-20 |
| BMI_positive | Positive control gene set for BMI | 7.75E-14 |
| rctm0401 | FGFR2 ligand binding and activation | 8.36E-09 |
| rctm1135 | Signaling by NOTCH1 HD Domain Mutants in Cancer | 1.53E-08 |
| rctm0104 | Amine-derived hormones | 1.83E-08 |
| rctm0885 | Post-Elongation Processing of the Transcript | 6.82E-08 |
| M2130 | Ether lipid metabolism | 1.29E-06 |
