# Supplementary material for: Multi-Omics Data Analysis Uncovers Molecular Networks and Gene Regulators for Metabolic Biomarkers
Source: Biomolecules. 2021 Mar 10;11(3):406. doi: 10.3390/biom11030406 (PMC8001935; doi:10.3390/biom11030406)
Supplement: Supplementary file 1 [file biomolecules-11-00406-s001.zip › Supple Figure 4.docx]

Figure S4. Comparison of significant pathways (false discovery rate [FDR] < 0.05) between insulin-like growth factor-I (IGF-I) and insulin resistance (IR) phenotypes (IGF-I/IR, 50-kb distance–based and expression quantitative trait loci [eQTL]–based mapping to genes; yellow-highlighted pathways are significant [FDR < 0.05] in the marker-set enrichment meta-analysis of IGF-I-eQTL and IR-eQTL)

**Six common pathways shared by IGF-I and IR**

**distance**–**based and eQTL**–**based pathways**

| **Pathway** | **Description** |
| --- | --- |
| M19708 | Type 2 diabetes mellitus |
| rctm0527 | HS-GAG biosynthesis |
| rctm0648 | Lipoprotein metabolism |
| rctm0709 | Mitochondrial Protein Import |
| rctm1114 | Signaling by EGFR |
| rctm1115 | Signaling by EGFR in Cancer |
