# Supplementary material for: Multi-Omics Data Analysis Uncovers Molecular Networks and Gene Regulators for Metabolic Biomarkers
Source: Biomolecules. 2021 Mar 10;11(3):406. doi: 10.3390/biom11030406 (PMC8001935; doi:10.3390/biom11030406)
Supplement: Supplementary file 1 [file biomolecules-11-00406-s001.zip › Supple Table 1.docx]

Table S1. Meta-MSEA analysis of IGF-I and IR pathways (IGF-I/IR, eQTL-based mapping to genes; pathways arranged by ascending FDR)

| **Module (n = 77)** | **Description** | **FDR < 0.05** |
| --- | --- | --- |
| rctm0449 | G alpha (s) signaling events | 9.14E-82 |
| rctm0424 | Formation of fibrin clot (clotting cascade) | 1.28E-79 |
| rctm1266 | The citric acid (TCA) cycle and respiratory electron transport | 8.62E-72 |
| M7761 | Melanogenesis | 2.09E-57 |
| M19540 | Oxidative phosphorylation | 5.57E-52 |
| rctm0066 | Activation of NMDA receptor upon glutamate binding and postsynaptic events | 5.11E-48 |
| M3494 | Thrombin signaling and protease-activated receptors | 5.61E-48 |
| M14314 | Purine metabolism | 1.02E-47 |
| M16817 | Oocyte meiosis | 6.76E-47 |
| M3578 | Progesterone-mediated oocyte maturation | 1.42E-46 |
| rctm0225 | Cell-cell junction organization | 1.22E-45 |
| rctm0224 | Cell-cell communication | 6.68E-42 |
| rctm0709 | Mitochondrial protein import | 1.68E-40 |
| rctm0882 | Post NMDA receptor activation events | 1.45E-39 |
| M16848 | Epithelial cell signaling in *Helicobacter pylori* infection | 1.50E-34 |
| rctm0222 | Cell junction organization | 2.27E-34 |
| rctm1046 | Respiratory electron transport, ATP synthesis by chemiosmotic coupling, and heat production by uncoupling proteins | 2.67E-34 |
| M7014 | PKC-catalyzed phosphorylation of inhibitory phosphoprotein of myosin phosphatase | 2.97E-34 |
| rctm0415 | Fatty acid, triacylglycerol, and ketone body metabolism | 1.33E-29 |
| M17906 | *Vibrio cholerae* infection | 1.40E-29 |
| rctm0862 | Phospholipase C-mediated cascade | 5.87E-29 |
| rctm0951 | RNA polymerase II transcription | 1.69E-28 |
| rctm0527 | HS-GAG biosynthesis | 1.63E-26 |
| rctm0613 | Interferon gamma signaling | 5.14E-25 |
| rctm0949 | RNA polymerase II pre-transcription events | 1.64E-24 |
| rctm0842 | PLCG1 events in ERBB2 signaling | 3.45E-24 |
| rctm0294 | DAG and IP3 signaling | 8.35E-23 |
| rctm0315 | Degradation of collagen | 1.14E-22 |
| rctm0437 | Formation of the ternary complex and, subsequently, the 43S complex | 1.19E-22 |
| rctm1293 | Transcription of the HIV genome | 1.59E-22 |
| rctm0355 | EGFR interacts with phospholipase C-gamma | 4.39E-22 |
| M7272 | Parkinson's disease | 8.35E-22 |
| rctm1045 | Respiratory electron transport | 2.66E-21 |
| rctm0384 | Eukaryotic translation initiation | 5.99E-21 |

Table S1 (Continued)

| **Module (n = 77)** | **Description** | **FDR < 0.05** |
| --- | --- | --- |
| M6382 | Regulation of autophagy | 8.05E-21 |
| rctm0840 | PLC-gamma1 signaling | 3.63E-20 |
| rctm0258 | Collagen biosynthesis and modifying enzymes | 1.05E-19 |
| rctm0211 | Cap-dependent translation initiation | 9.40E-19 |
| rctm0331 | Disease | 2.77E-18 |
| M5109 | Pyrimidine metabolism | 1.53E-17 |
| rctm1058 | Rho GTPase cycle | 1.54E-17 |
| rctm1144 | Signaling by rho GTPases | 1.54E-17 |
| rctm0497 | Glucagon signaling in metabolic regulation | 4.98E-17 |
| M7330 | Glycosaminoglycan biosynthesis – heparan sulfate | 1.78E-16 |
| rctm0063 | Activation of matrix metalloproteinases | 2.46E-16 |
| rctm0310 | Deadenylation-dependent mRNA decay | 4.52E-16 |
| rctm0428 | Formation of a pool of free 40S subunits | 1.27E-15 |
| rctm0317 | Degradation of the extracellular matrix | 3.12E-15 |
| M2890 | Calcium signaling pathway | 4.92E-15 |
| rctm0627 | Iron uptake and transport | 7.06E-15 |
| M14091 | Olfactory transduction | 1.17E-14 |
| rctm1114 | Signaling by EGFR | 1.17E-14 |
| rctm0635 | L13a-mediated translational silencing of ceruloplasmin expression | 4.09E-14 |
| rctm0683 | Metabolism of amino acids and derivatives | 6.49E-14 |
| rctm0596 | Insulin receptor recycling | 6.53E-14 |
| rctm0003 | 3' -UTR-mediated translational regulation | 6.61E-14 |
| rctm1115 | Signaling by EGFR in cancer | 7.28E-14 |
| rctm0482 | GTP hydrolysis and joining of the 60S ribosomal subunit | 7.34E-14 |
| rctm0450 | G alpha (z) signaling events | 1.19E-13 |
| rctm0759 | NRAGE signals death through JNK | 5.68E-13 |
| rctm0800 | Nuclear signaling by ERBB4 | 1.20E-12 |
| rctm1301 | Transferrin endocytosis and recycling | 2.81E-12 |
| rctm0857 | Phagosomal maturation (early endosomal stage) | 6.98E-12 |
| rctm1170 | Sphingolipid metabolism | 7.23E-12 |
| rctm0641 | Latent infection of *Homo sapiens* with *Mycobacterium tuberculosis* | 1.54E-11 |
| M9131 | Glycerophospholipid metabolism | 4.21E-11 |
| rctm0111 | Amyloids | 4.28E-11 |
| rctm0514 | Glycosphingolipid metabolism | 5.75E-11 |
| rctm0475 | GPCR downstream signaling | 2.75E-10 |
| M16473 | Aldosterone-regulated sodium reabsorption | 7.05E-10 |

Table S1 (Continued)

| **Module (n = 77)** | **Description** | **FDR < 0.05** |
| --- | --- | --- |
| rctm1228 | TCR signaling | 9.11E-10 |
| rctm1302 | Translation | 1.05E-09 |
| rctm1128 | Signaling by GPCR | 2.26E-09 |
| rctm1118 | Signaling by FGFR | 5.37E-09 |
| rctm1119 | Signaling by FGFR in disease | 2.44E-06 |
| M9387 | Vascular smooth muscle contraction | 5.92E-06 |
| M19708 | Type 2 diabetes mellitus | 2.74E-05 |
| rctm1388 | mRNA 3'-end processing | 7.17E-05 |
| rctm0883 | Post-elongation processing of intron-containing pre-mRNA | 0.00010396 |

FDR, false discovery rate; eQTL, expression quantitative trait loci; IGF-I, insulin-like growth factor-I; IR, insulin resistance; MSEA, marker-set enrichment analysis.
