# Supplementary material for: Multi-Omics Data Analysis Uncovers Molecular Networks and Gene Regulators for Metabolic Biomarkers
Source: Biomolecules. 2021 Mar 10;11(3):406. doi: 10.3390/biom11030406 (PMC8001935; doi:10.3390/biom11030406)
Supplement: Supplementary file 1 [file biomolecules-11-00406-s001.zip › Supple Table 2.docx]

Table S2. IGF-I and IR pathways (eQTL-based mapping to genes) from the MSEA meta-analysis and corresponding tissue-specific network key drivers

|  |  |  | **Top 5 key drivers** | | | | |
| --- | --- | --- | --- | --- | --- | --- | --- |
| **Module** | **Description** | **Module size** | **Adipose** | **Blood** | **Liver** | **Muscle** | **PPI** |
| M14314 | Purine metabolism | N/A, N/A, N/A, N/A, 33§ | N/A | N/A | N/A | N/A | *ZNRD1*, POLR2H*, POLR2L*, POLR1E*, POLR3C* |
| M16473 | Aldosterone-regulated sodium reabsorption | N/A, N/A, N/A, N/A, 15§ | N/A | N/A | N/A | N/A | *IRS1*, TNK1, TYK2, JAK1, FRAP1* |
| M16817 | Oocyte meiosis | N/A, N/A, N/A, N/A, 43§ | N/A | N/A | N/A | N/A | *PPP2CA*, CDK2*, PRKCB1, CAMK2A, PRKCG* |
| M16848 | Epithelial cell signaling in *Helicobacter pylori* infection | N/A, N/A, N/A, N/A, 29§ | N/A | N/A | N/A | N/A | *AKT1, LCK, EGFR, PRKCA, ATP6V1H* |
| M17906 | *Vibrio cholerae* infection | N/A, N/A, N/A, N/A, 32§ | N/A | N/A | N/A | N/A | *ATP6V1D*, ATP6V1E1*, ATP6V0A1*, ATP6V1C1*, ATP6V1H* |
| M19708 | Type 2 diabetes mellitus | N/A, N/A, N/A, N/A, 17§ | N/A | N/A | N/A | N/A | *IRS1*, HRAS, JAK1, IGF1R, AKT1* |
| M2890 | Calcium signaling pathway | N/A, N/A, N/A, N/A, 59§ | N/A | N/A | N/A | N/A | *PRKACA*, GNA11*, PRKCG, PRKACB, PRKCB1* |

| Table S2. (Continued) | | | | | | | |
| --- | --- | --- | --- | --- | --- | --- | --- |
|  |  |  | **Top 5 key drivers** | | | | |
| **Module** | **Description** | **Module size** | **Adipose** | **Blood** | **Liver** | **Muscle** | **PPI** |
| M3494 | Thrombin signaling and protease-activated receptors | N/A, N/A, N/A, N/A, 18§ | N/A | N/A | N/A | N/A | *RHOA, YWHAG, RAC1, SRRM2* |
| M3578 | Progesterone-mediated oocyte maturation | N/A, N/A, N/A, N/A, 32§ | N/A | N/A | N/A | N/A | *CDK2*, PLK1*, AURKA, AURKB, PDPK1* |
| M7761 | Melanogenesis | N/A, N/A, N/A, N/A, 49§ | N/A | N/A | N/A | N/A | *PRKCB1, RPS6KA3, PRKCG, MAP2, CTNNB1* |
| M9387 | Vascular smooth muscle contraction | N/A, N/A, N/A, N/A, 71§ | N/A | N/A | N/A | N/A | *HRAS*, MAP2K1*, PRKCG, CAMK2G, CAMK2B* |
| rctm0003 | 3'-UTR-mediated translational regulation | 40**, 29¶, N/A, N/A, 38§ | *RPL31*, FAU*, RPL27*, RPS3A*, RPL13A* | *RPL9** | N/A | N/A | *GSPT2, RPL41, RPS4Y1, RPL3L, RPL36A* |
| rctm0066 | Activation of NMDA receptor upon glutamate binding and postsynaptic events | N/A, N/A, N/A, N/A, 12§ | N/A | N/A | N/A | N/A | *RPS6KA3, MAP2, PRKCB1, GSK3B, CAMK2A* |
| rctm0111 | Amyloids | N/A, N/A, N/A, N/A, 14§ | N/A | N/A | N/A | N/A | *YWHAE, NSFL1C* |
| rctm0224 | Cell-cell communication | N/A, N/A, N/A, N/A, 46§ | N/A | N/A | N/A | N/A | *RAC1*, PTK2B*, PIK3R1, CTNNB1, PTPN5* |
| rctm0317 | Degradation of the extracellular matrix | 25**, N/A, N/A, N/A, N/A | *CTSG** | N/A | N/A | N/A | *N/A* |
| rctm0331 | Disease | 346**, 199¶, N/A, N/A, 356§ | *FAU*, RPL10A*, RPL31*, MYO1F, ZFYVE28* | RPS18* | N/A | N/A | *PSMB9*, GTF2A2*, CTDP1*, PSMB8*, RPL36AL* |
| rctm0415 | Fatty acid, triacylglycerol, and ketone body metabolism | N/A, N/A, N/A, N/A, 46§ | N/A | N/A | N/A | N/A | *MED24*, MED15*, MED6*, MED1, CDK8* |
| rctm0475 | GPCR downstream signaling | 173**, N/A, 171¥, N/A, 162§ | *PKIB, MMT00035939, LGMN, IRF5, GLIPR1* | N/A | *HCK* | N/A | *GNA15*, GNA11*, GNAQ, GNA14, RHOA* |
| rctm0613 | Interferon gamma signaling | 25**, N/A, N/A, N/A, 21§ | *RTP4* | N/A | N/A | N/A | *JAK2*, TYK2* |
| rctm0683 | Metabolism of amino acids and derivatives | 93**, N/A, 86¥, N/A, 89§ | *HIBADH*, MCCC1*, ACADSB*, HIBCH*, CCBL2* | N/A | *ASL*, CPS1, ASS1* | N/A | *PSMB2*, PSMD10, PSMD12, PSMD6, PSMB6* |
| rctm1046 | Respiratory electron transport, ATP synthesis by chemiosmotic coupling, and heat production by uncoupling proteins. | 42**, N/A, N/A, N/A, 44§ | *NDUFB9*, SDHB*, COX4I1*, CYC1, FH* | N/A | N/A | N/A | *NDUFS2*, NDUFA8, NDUFA5, NDUFS1, NDUFS3* |
| rctm1058 | Rho GTPase cycle | N/A, N/A, N/A, N/A, 54§ | N/A | N/A | N/A | N/A | *CDC42*, RAC1, RHOA, PIK3R1, GDI1* |
| rctm1228 | TCR signaling | 20**, N/A, N/A, N/A, 21§ | *LCK* | N/A | N/A | N/A | *LCK, LYN, FYN, SYK, ZAP70* |
| rctm1293 | Transcription of the HIV genome | N/A, N/A, N/A, N/A, 37§ | N/A | N/A | N/A | N/A | *POLR2J*, GTF2F2*, POLR2K, POLR2F, MNAT1* |

eQTL, expression quantitative trait loci; IGF-I, insulin-growth factor-I; IR, insulin resistance; MSEA, marker-set enrichment analysis; N/A, not available; PPI, protein to protein interaction network.

** Number of genes in adipose-specific network pathways.

¶ Number of genes in blood-specific network pathways.

¥ Number of genes in liver-specific network pathways.

† Number of genes in muscle-specific network pathways.

§ Number of genes in PPI-based network pathways.

* Member gene of the particular pathway in tissue-specific gene-regulatory network analysis.
