# Supplementary material for: Multi-Omics Data Analysis Uncovers Molecular Networks and Gene Regulators for Metabolic Biomarkers
Source: Biomolecules. 2021 Mar 10;11(3):406. doi: 10.3390/biom11030406 (PMC8001935; doi:10.3390/biom11030406)
Supplement: Supplementary file 1 [file biomolecules-11-00406-s001.zip › Supple Table 3.docx]

Table S3. IR pathways (eQTL-based mapping to genes) from MSEA and corresponding tissue-specific network key drivers

|  |  |  | **Top 5 key drivers** | | | | |
| --- | --- | --- | --- | --- | --- | --- | --- |
| **Module** | **Description** | **Module size** | **Adipose** | **Blood** | **Liver** | **Muscle** | **PPI** |
| M10082 | TNFR2 signaling pathway | N/A, N/A, N/A, N/A, 10§ | N/A | N/A | N/A | N/A | *TUBA3D, HSP90AA2, HSPA1L, IKBKB, HSPA1B* |
| M10462 | Adipocytokine signaling pathway | N/A, N/A, N/A, N/A, 33§ | N/A | N/A | N/A | N/A | *GSK3B, FRAP1, HSP90AA2, PDPK1, IKBKB* |
| M10792 | MAPK signaling pathway | N/A, N/A, N/A, N/A, 63§ | N/A | N/A | N/A | N/A | *MAPK9*, MAPK8*, MAP2K1*, MAP3K11*, MAPK10* |
| M11673 | Biosynthesis of unsaturated fatty acids | N N/A, N/A, 14¥, N/A, N/A | N/A | N/A | *ELOVL5** | N/A | N/A |
| M11675 | Homologous recombination | N/A, N/A, N/A, N/A, 16§ | N/A | N/A | N/A | N/A | *TOP3A*, RPA2*, RPA3*, PCNA, RAD51* |
| M11835 | Valine, leucine, and isoleucine degradation | 36**, N/A, 35¥, N/A, N/A | *ECHS1*, MCCC1*, ACAT1*, HADH*, CCBL2* | N/A | *ACADM*, ACSM3* | N/A | N/A |
| M14314 | Purine metabolism | N/A, N/A, N/A, N/A, 37§ | N/A | N/A | N/A | N/A | *POLR2H*, ZNRD1*, POLR1E*, POLR2K, POLR3C* |

| Table S3. (continued) | | | | | | | |
| --- | --- | --- | --- | --- | --- | --- | --- |
|  |  |  | **Top 5 key drivers** | | | | |
| **Module** | **Description** | **Module size** | **Adipose** | **Blood** | **Liver** | **Muscle** | **PPI** |
| M1519 | Endocytosis | N/A, N/A, N/A, N/A, 82§ | N/A | N/A | N/A | N/A | *CBL*, EGF*, SH3GL2, RNF11, CAV1* |
| M1547 | Control of skeletal myogenesis by HDAC and calcium/calmodulin-dependent kinase (CaMK) | N/A, N/A, N/A, N/A, 14§ | N/A | N/A | N/A | N/A | *MAPK14*, PRKCA*, CALM3*, SRC, PTK2B* |
| M16120 | How does *Salmonella* hijack a cell | N/A, N/A, N/A, N/A, 14§ | N/A | N/A | N/A | N/A | *WASL*, ACTR3*, WAS, FLNA, RPLP0* |
| M16473 | Aldosterone-regulated sodium reabsorption | N/A, N/A, N/A, N/A, 16§ | N/A | N/A | N/A | N/A | *IRS1*, TNK1, TYK2, JAK1, FRAP1* |
| M16476 | Cell adhesion molecules (CAMs) | N/A, N/A, N/A, N/A, 47§ | N/A | N/A | N/A | N/A | *FN1, CSK* |
| M16517 | WNT signaling pathway | N/A, N/A, N/A, N/A, 10§ | N/A | N/A | N/A | N/A | *DVL1, CTNNB1* |
| M16817 | Oocyte meiosis | N/A, N/A, N/A, N/A, 44§ | N/A | N/A | N/A | N/A | *CDK2*, PPP2CA*, CAMK2A, PRKCB1, PRKCG* |
| M16848 | Epithelial cell signaling in *Helicobacter pylori* infection | N/A, N/A, N/A, N/A, 31§ | N/A | N/A | N/A | N/A | *ATP6V1E1*, ATP6V0D1*, AKT1, FOS, ATP6V1H* |
| M16853 | DNA replication | N/A, N/A, N/A, N/A, 26§ | N/A | N/A | N/A | N/A | *RPA1*, PCNA*, RPA2*, POLD1, POLE* |
| M16894 | Complement and coagulation cascades | N/A, N/A, N/A, N/A, 30§ | N/A | N/A | N/A | N/A | *C4A*, F2, IGHV4-31, FGA, FN1* |
| M17906 | *Vibrio cholerae* infection | N/A, N/A, N/A, N/A, 21§ | N/A | N/A | N/A | N/A | *ATP6V1D*, ATP6V1E1*, ATP6V0A1*, ATP6V1C1*, ATP6V1H* |
| M18155 | Insulin signaling pathway | N/A, N/A, N/A, N/A, 58§ | N/A | N/A | N/A | N/A | *IRS1*, HRAS*, RAC1, JAK1, RPS6KA3* |
| M18306 | Regulation of actin cytoskeleton | N/A, N/A, N/A, N/A, 98§ | N/A | N/A | N/A | N/A | *ROCK2*, HRAS*, CDC42*, PIK3CA, WAS* |
| M1940 | Regulation and function of ChREBP in liver | N/A, N/A, N/A, N/A, 27§ | N/A | N/A | N/A | N/A | *PPP2R1A*, PPP2CA*, CLTC, RAF1, MYH10* |
| M19428 | Wnt signaling pathway | N/A, N/A, N/A, N/A, 61§ | N/A | N/A | N/A | N/A | *DVL3*, PPP2CA*, DVL1, DVL2, CTNNB1* |
| M19708 | Type 2 diabetes mellitus | N/A, N/A, N/A, N/A, 17§ | N/A | N/A | N/A | N/A | *IRS1*, HRAS, JAK1, IGF1R, AKT1* |
| M2333 | Pathogenic *Escherichia coli* infection | N/A, N/A, N/A, N/A, 32§ | N/A | N/A | N/A | N/A | *CFL1, CAPZA2, LIMA1, MYL6, GSN* |
| M2499 | CARM1 and regulation of the estrogen receptor | N/A, N/A, N/A, N/A, 13§ | N/A | N/A | N/A | N/A | *GTF2B* |
| M2890 | calcium signaling pathway | N/A, N/A, N/A, N/A, 57§ | N/A | N/A | N/A | N/A | *GNA11*, PRKCG, PRKACA, GNAQ, PRKCB1* |
| M3115 | Long-term potentiation | N/A, N/A, N/A, N/A, 26§ | N/A | N/A | N/A | N/A | *PRKCA*, PRKCB1, RPS6KA3, CAMK2A, PRKCG* |
| M3494 | Thrombin signaling and protease-activated receptors | N/A, N/A, N/A, N/A, 17§ | N/A | N/A | N/A | N/A | *RHOA, YWHAG, RAC1, SRRM2* |
| M3578 | Progesterone-mediated oocyte maturation | N/A, N/A, N/A, N/A, 30§ | N/A | N/A | N/A | N/A | *CDK2*, PLK1*, AURKB, AURKA, TNK1* |
| M4086 | Propanoate metabolism | 26**, N/A, 26¥, N/A, N/A | *PCCA*, DBT, MCCC1, HADH* | N/A | *ACADM** | N/A | N/A |
| M4791 | Regulation of eIF4e and p70 S6 Kinase | N/A, N/A, N/A, N/A, 16§ | N/A | N/A | N/A | N/A | *MAPK1*, AKT1*, FRAP1, CSNK2A1P, RAC1* |
| M6220 | Agrin in postsynaptic differentiation | N/A, N/A, N/A, N/A, 16§ | N/A | N/A | N/A | N/A | *RAC1*, MAPK1*, SRC, ARHGEF7, MAP2K1* |
| M648 | Cell cycle: G1/S check point | N/A, N/A, N/A, N/A, 10§ | N/A | N/A | N/A | N/A | *CDK2*, CDC2* |
| M699 | Fatty acid metabolism | 30**, N/A, 30¥, 28†, N/A | *HADHB*, ACADVL*, ECHS1*, ETFDH* | N/A | *HADH*, ACADM** | *HADHB** | N/A |
| M7761 | Melanogenesis | N/A, N/A, N/A, N/A, 37§ | N/A | N/A | N/A | N/A | *GSK3B*, CAMK2A, RPS6KA3, CTNNB1, DLG4* |
| M835 | Dilated cardiomyopathy | N/A, N/A, N/A, N/A, 41§ | N/A | N/A | N/A | N/A | *ITGB1, CSK, FN1, CTNNB1, PXN* |
| M8719 | mCalpain and friends in cell motility | N/A, N/A, N/A, N/A, 12§ | N/A | N/A | N/A | N/A | *PTPN1, PIK3CA, FGD4, ARC, CACNG2* |
| M8731 | Aspirin blocks signaling pathway involved in platelet activation | N/A, N/A, N/A, N/A, 14§ | N/A | N/A | N/A | N/A | *PLCB1*, MAPK3*, RPS6KA3, PRKCB1, PTK2B* |
| M9387 | Vascular smooth muscle contraction | N/A, N/A, N/A, N/A, 69§ | N/A | N/A | N/A | N/A | *HRAS*, CAMK2G, PRKCG, CAMK2B, PRKCD* |
| M963 | RNA degradation | N/A, N/A, N/A, N/A, 30§ | N/A | N/A | N/A | N/A | *DDX6*, DCP1A*, XRN1, RPL27, ATP6V1B2* |
| M9904 | T cell–receptor signaling pathway | N/A, N/A, N/A, N/A, 60§ | N/A | N/A | N/A | N/A | *AKT1*, LCK, RAC1, VAV1, JAK1* |
| rctm0089 | Adaptive immune system | N/A, N/A, N/A, N/A, 113§ | N/A | N/A | N/A | N/A | *UBE2D1*, UBE2D3, UBE2D2, UBE2E1, RBX1* |
| rctm0111 | Amyloids | N/A, N/A, N/A, N/A, 16§ | N/A | N/A | N/A | N/A | *ATP5O* |
| rctm0168 | Binding and uptake of ligands by scavenger receptors | N/A, N/A, N/A, N/A, 33§ | N/A | N/A | N/A | N/A | *IGHV4-31, C1QC, IGHV, C1QA* |
| rctm0181 | Budding and maturation of HIV virion | N/A, N/A, N/A, N/A, 14§ | N/A | N/A | N/A | N/A | *VPS4B** |
| rctm0211 | Cap-dependent translation initiation | 69**, 40¶, N/A, N/A, 69§ | *RPL31*, FAU*, RPL10A** | *RPLP2*, RPL9*, RPS18** | N/A | N/A | *FAU*, ETF1*, RPS21, RPS4Y1, RPL26L1* |
| rctm0218 | Cell cycle | 186**, N/A, 174¥, N/A, 200§ | *DBF4*, CDCA8, FANCI, SPAG5, MELK* | N/A | *MCM6*, CDCA8* | N/A | *PAFAH1B1*, MAD1L1*, NDE1*, NUP37, SGOL1* |
| rctm0223 | Cell surface interactions at the vascular wall | N/A, N/A, 41¥, N/A, 49§ | N/A | N/A | *FCER1G*, APBB1IP, FERMT3* | N/A | *FN1, CSK, ITGB1, PIK3R1, PIK3R2* |
| rctm0224 | Cell-cell communication | N/A, N/A, N/A, N/A, 55§ | N/A | N/A | N/A | N/A | *RAC1*, PIK3R1, CTNNB1, PTPN5, DUSP4* |
| rctm0238 | Chromosome maintenance | N/A, N/A, N/A, N/A, 36§ | N/A | N/A | N/A | N/A | *PCNA*, RPA1*, RPA2*, RFC2*, POLE* |
| rctm0261 | Complement cascade | N/A, N/A, N/A, N/A, 28§ | N/A | N/A | N/A | N/A | *C1QA*, IGHV4-31, C1QC, IGHV* |
| rctm0274 | Cooperation of prefoldin and TriC/CCT  in actin and tubulin folding | N/A, N/A, N/A, N/A, 14§ | N/A | N/A | N/A | N/A | *TUBA4A*, CCT6A*, MAP3K1, NOL5A, YWHAE* |
| rctm0275 | Costimulation by the CD28 family | N/A, N/A, N/A, N/A, 35§ | N/A | N/A | N/A | N/A | *AKT1*, PPP2CA*, PPP2R1A*, RAC1*, PDPK1* |
| rctm0289 | Cytokine signaling in immune system | 51**, N/A, N/A, 44†, 57§ | *RTP4* | N/A | N/A | *RTP4* | *SEH1L*, HMGA1, NUP62, NUP133, NUP153* |
| rctm0297 | DARPP-32 events | N/A, N/A, N/A, N/A, 18§ | N/A | N/A | N/A | N/A | *CALM3*, PPP2CA*, CLTC, PRKCB1, PRKACA* |
| rctm0303 | DNA repair | N/A, N/A, N/A, N/A, 62§ | N/A | N/A | N/A | N/A | *GTF2H1*, GTF2H2*, ERCC2, VARS2, GTF2H3* |
| rctm0304 | DNA replication | N/A, N/A, N/A, N/A, 41§ | N/A | N/A | N/A | N/A | *PSMD13*, PSMC5*, PSMC1*, PSMD8*, CDC6* |
| rctm0324 | Destabilization of mRNA by butyrate response factor 1 (BRF1) | N/A, N/A, N/A, N/A, 12§ | N/A | N/A | N/A | N/A | *SKIV2L2* |
| rctm0331 | Disease | 358**, 198¶, N/A, N/A, 358§ | *RPL31*, FAU*, MYO1F* | *RPS18** | N/A | N/A | *PSMB9*, GTF2A2*, PSMF1*, CTDP1*, RPL36AL* |
| rctm0350 | E2F-mediated regulation of DNA replication | N/A, N/A, N/A, N/A, 12§ | N/A | N/A | N/A | N/A | *CDC2, CDK2* |
| rctm0354 | EGFR downregulation | N/A, N/A, N/A, N/A, 15§ | N/A | N/A | N/A | N/A | *EGF*, UBA52*, EGFR, UBC, RPS27A* |
| rctm0392 | FCERI mediated Ca^+2^ mobilization | N/A, N/A, N/A, N/A, 47§ | N/A | N/A | N/A | N/A | *PIK3R1, PLCG1, IGHV@, C1QA, IGHV4-31* |
| rctm0411 | Factors involved in megakaryocyte development and platelet production | N/A, N/A, N/A, N/A, 42§ | N/A | N/A | N/A | N/A | *PRKAR1A*, IRS1, BAD* |
| rctm0415 | Fatty acid, triacylglycerol, and ketone body metabolism | 80**, N/A, 79¥, N/A, 74§ | *ECHS1*, FASN*, ACSL1*, THRSP, CPT2* | N/A | *ELOVL6** | N/A | *MED24*, MED15*, MED1, NUMA1, HCFC1* |
| rctm0418 | Fcgamma receptor (FCGR)–dependent phagocytosis | N/A, N/A, N/A, N/A, 54§ | N/A | N/A | N/A | N/A | *WASL*, RAC1*, WAS, IQGAP1, SOS1* |
| rctm0449 | G alpha (s) signaling events | N/A, N/A, N/A, N/A, 37§ | N/A | N/A | N/A | N/A | *GNAS*, GNB2, GNB4, GNB1, GNG12* |
| rctm0450 | G alpha (z) signaling events | N/A, N/A, N/A, N/A, 22§ | N/A | N/A | N/A | N/A | *GNAS*, GNB4, GNB2, GNB1, GNG12* |
| rctm0463 | G2/M checkpoints | N/A, N/A, N/A, N/A, 23§ | N/A | N/A | N/A | N/A | *RPA3*, RPA2*, RPA1*, MCM7, PCNA* |
| rctm0477 | GPVI-mediated activation cascade | N/A, N/A, N/A, N/A, 16§ | N/A | N/A | N/A | N/A | *PIK3R5*, PIK3R1, VAV1, PIK3CD, PIK3CB* |
| rctm0531 | Hemostasis | 203**, N/A, 194¥, 178†, 215§ | *CD84*, FERMT3, IRF5, ATF3, MYO1F* | N/A | *APBB1IP*, FCER1G*, NCKAP1L, PTPRC, FERMT3* | *PTPN6** | *VAV1, BMX, PIK3CB, ARC, PIK3C2B* |
| rctm0591 | Innate immune system | 251**, N/A, 252¥, 223†, 282§ | *LAT2*, PTPN6, NCKAP1L, IL10RA, IRF5* | N/A | *TYROBP*, NCKAP1L, RAC2, NCF2, IGSF6* | *AK014135, COTL1* | *GRB2*, MAPKAPK2, RAP2A, FRK, C1QC* |
| rctm0598 | Integration of energy metabolism | N/A, N/A, N/A, N/A, 54§ | N/A | N/A | N/A | N/A | *PRKACA, PRKACB, PRKCB1, PRKCG, RAC1* |
| rctm0612 | Interferon alpha/beta signaling | N/A, N/A, 23¥, N/A, N/A | N/A | N/A | ISG15* | N/A | N/A |
| rctm0618 | Interleukin-3, 5 and GM-CSF signaling | N/A, N/A, N/A, N/A, 20§ | N/A | N/A | N/A | N/A | *SHC1*, PIK3R1, SRC, EGFR, VAV1* |
| rctm0636 | L1CAM interactions | N/A, N/A, N/A, N/A, 37§ | N/A | N/A | N/A | N/A | *MAPK3*, PAK4, CDK5, RPS6KA3, CSK* |
| rctm0656 | M phase | 38**, N/A, N/A, N/A, 45§ | *CDCA8* | N/A | N/A | N/A | *SEH1L*, SGOL2, SGOL1, ERCC6L, KIF18A* |
| rctm0683 | Metabolism of amino acids and derivatives | 102**, N/A, 93¥, N/A, 98§ | *ACADSB*, MCCC1*, HIBADH*, HIBCH*, CCBL2* | N/A | *ASL*, CPS1, ASS1* | N/A | *PSMD10, PSMD12, PSMB7, PSMB6, PSME2* |
| rctm0686 | Metabolism of lipids and lipoproteins | 254**, N/A, 259¥, 222†, 229§ | *ECHS1*, FASN*, ACSL1*, ACAT1*, EHHADH* | N/A | *ELOVL5*, PEX11A*, STARD4*, FDFT1*, ALDH3A2* | *SLC36A2* | *PTMA, UCHL5IP, TREX2, LEPRE1, RECQL5* |
| rctm0689 | Metabolism of non-coding RNA | N/A, N/A, N/A, N/A, 32§ | N/A | N/A | N/A | N/A | *SEH1L*, HMGA1, NUP54, RANP1, NUP62* |
| rctm0707 | Mitochondrial fatty acid beta-oxidation | 11**, N/A, N/A, N/A, N/A | *ECHS1*, MCCC1, CPT2* | N/A | N/A | N/A | N/A |
| rctm0721 | Mitotic prophase | N/A, N/A, N/A, N/A, 37§ | N/A | N/A | N/A | N/A | *RANP1, RANBP2, NUP133, NUP62, NUP153* |
| rctm0732 | MyD88 cascade initiated on plasma membrane | N/A, N/A, N/A, N/A, 46§ | N/A | N/A | N/A | N/A | *MAPK14*, MAPK8*, GSK3B, TRAF6, PRKACA* |
| rctm0770 | Negative regulation of FGFR signaling | N/A, N/A, N/A, N/A, 16§ | N/A | N/A | N/A | N/A | *MAPK1*, PTPN11, CLTC, CDC2, SRC* |
| rctm0798 | Nuclear receptor transcription pathway | N/A, N/A, N/A, N/A, 13§ | N/A | N/A | N/A | N/A | *NCOA1, NCOR1* |
| rctm0875 | Platelet homeostasis | N/A, N/A, N/A, N/A, 44§ | N/A | N/A | N/A | N/A | *PPP2R1B*, PPP2CA*, GNB4, PRKACA, NME2* |
| rctm0876 | Platelet sensitization by LDL | N/A, N/A, N/A, N/A, 20§ | N/A | N/A | N/A | N/A | *PPP2CA*, PPP2R1A*, PPP2R1B*, GSK3B*, MAPK1* |
| rctm0882 | Post NMDA receptor activation events | N/A, N/A, N/A, N/A, 12§ | N/A | N/A | N/A | N/A | *RPS6KA3, MAP2, PRKCB1, GSK3B, CAMK2A* |
| rctm0987 | Regulation of cholesterol biosynthesis by SREBP (SREBF) | N/A, N/A, 23¥, N/A, N/A | N/A | N/A | *ACSS2* | N/A | N/A |
| rctm1046 | Respiratory electron transport, ATP synthesis by chemiosmotic coupling, and heat production by uncoupling proteins. | 48**, N/A, N/A, N/A, 52§ | *NDUFB9*, COX4I1*, NDUFV2*, CYC1, FH* | N/A | N/A | N/A | *NDUFS2*, NDUFV2*, NDUFS3, NDUFA5, NDUFA8* |
| rctm1058 | Rho GTPase cycle | N/A, N/A, N/A, N/A, 54§ | N/A | N/A | N/A | N/A | *CDC42*, RAC1, RHOA, PIK3R1, GDI1* |
| rctm1109 | Signal attenuation | N/A, N/A, N/A, N/A, 14§ | N/A | N/A | N/A | N/A | *SHC1*, INSR*, IRS1*, PIK3R1, SRC* |
| rctm1117 | Signaling by ERBB4 | N/A, N/A, N/A, N/A, 49§ | N/A | N/A | N/A | N/A | *AKT1*, HRAS*, IRS1*, AKT2*, MAP2K1** |
| rctm1150 | Signaling by Wnt | N/A, N/A, N/A, N/A, 95§ | N/A | N/A | N/A | N/A | *PSMB2*, PSMB7, PSMB6, PSMD12, PSMD10* |
| rctm1228 | TCR signaling | N/A, N/A, N/A, N/A, 20§ | N/A | N/A | N/A | N/A | *LCK, LYN, FYN, VAV1, SYK* |
| rctm1395 | mRNA processing | N/A, N/A, N/A, N/A, 47§ | N/A | N/A | N/A | N/A | *SNRPF*, SNRPE*, SFRS3, NCBP2, PABPN1* |

eQTL, expression quantitative trait loci; IR, insulin resistance; MSEA, marker-set enrichment analysis; N/A, not available; PPI, protein to protein interaction network.

** Number of genes in adipose-specific network pathways.

¶ Number of genes in blood-specific network pathways.

¥ Number of genes in liver-specific network pathways.

† Number of genes in muscle-specific network pathways.

§ Number of genes in PPI-based network pathways.

* Member gene of the particular pathway in tissue-specific gene-regulatory network analysis.
